# Mucosal Immune Responses Against an Oral Enterotoxigenic *Escherichia coli* Vaccine Evaluated in Clinical Trials

**DOI:** 10.1093/infdis/jiab475

**Published:** 2021-09-22

**Authors:** Ann-Mari Svennerholm, Anna Lundgren, Susannah Leach, Marjahan Akhtar, Firdausi Qadri

**Affiliations:** 1 Department of Microbiology and Immunology, University of Gothenburg, Gothenburg, Sweden; 2 Department of Clinical Pharmacology, Sahlgrenska University Hospital, Gothenburg, Sweden; 3 Infectious Diseases Division of icddr,b, Dhaka, Bangladesh

**Keywords:** ETEC, mucosal immune responses, vaccine

## Abstract

Enterotoxigenic *Escherichia coli* (ETEC) is a leading cause of mortality and morbidity in children in low-income countries. We have tested an oral ETEC vaccine, ETVAX, consisting of inactivated *E coli* overexpressing the most prevalent colonization factors and a toxoid, LCTB*A*, administered together with a mucosal adjuvant, double-mutant heat-labile toxin (dmLT), for capacity to induce mucosal immune responses and immunological memory against the primary vaccine antigens, ie, colonization factors, heat-labile toxin B-subunit and O antigen. The studies show that ETVAX could induce strong intestine-derived and/or fecal immune responses in a majority of vaccinated Swedish adults and in different age groups, including infants, in Bangladesh.

Enterotoxigenic *Escherichia coli* (ETEC) bacteria are a common cause of diarrhea in children in low- and middle-income countries. ETEC are heterogeneous enteric pathogens expressing 1 or more of at least 25 different colonization factors (CFs) and producing heat-labile toxin (LT) or heat-stable toxin (ST) or both toxins; some of the CFs are more prevalent than others [1, 2]. After initial development and testing of an oral ETEC vaccine consisting of inactivated ETEC bacteria and cholera toxin B subunit (CTB) [3], we have developed an improved second-generation oral ETEC vaccine (ETVAX) [4, 6]. ETVAX consists of 4 recombinant *E coli* strains overexpressing the most prevalent ETEC CFs, ie, CFA/I, CS3, CS5, and CS6, at significantly higher levels than expressed by clinical ETEC isolates, and an LT-like toxoid, a hybrid molecule of the B subunits of CT and LT, LCTB*A* [3, 4]. In previous animal studies, we have demonstrated that the immunogenicity of ETVAX protein antigens benefit from combination with a mucosal adjuvant, the double-mutant heat-labile toxin (dmLT) [5], to further enhance immunogenicity [4, 6].

ETVAX has been extensively tested for safety and capacity to induce mucosal immune responses in different target groups, ie, Swedish and Bangladeshi adults and descending age groups of Bangladeshi children [6, 7, 8]. Studies have also been undertaken to evaluate ETVAX for safety and immune responses in Phase 1 studies in young children and infants in Zambia. The primary objectives of the different studies have been to determine safety and mucosal, ie, intestinal or intestine-derived, immune responses as well as immunological memory induced by ETVAX against the primary vaccine antigens.

## MATERIALS AND METHODS

All studies were performed in accordance with the Declaration of Helsinki and approved by the relevant Ethical Review Committees. Written informed consent was obtained from all participants or parents of participants.

### Vaccine

The ETVAX vaccine used in the Swedish studies was produced for Scandinavian Biopharma by Unitech Biopharma (Matfors, Sweden) and for the Bangladeshi study by Biovian Oy (Turku, Finland). A full vaccine dose consists of 4 inactivated recombinant *E coli* strains (1 × 10^11^ bacteria) expressing high amounts of CFA/I, CS3, CS5, and CS6 and mixed with 1 mg of LCTB*A* toxoid; 3 of the vaccine strains are O78-positive *E coli*. The dmLT (R192G/L211A; WRAIR, Silver Spring, MD) in different dosages was used as adjuvant.

### Study Groups

The different study groups are summarized in Table 1.

#### Adults

This report reviews the mucosal immune responses determined in 129 adult Swedes and 45 adult Bangladeshis 18–45 years of age participating in 2 ETEC trials in Sweden and 1 trial in Bangladesh [6, 8, 9]. Different study groups of each population were given 2 oral doses 14 days apart from, respectively, ETVAX alone, ETVAX together with 10 µg of dmLT, or placebo in a double-blind manner; Swedish volunteers also received ETVAX + 25 µg of dmLT [6]. Immune responses were recorded before and 7 days after the first dose and 5–7 days after the second dose. A subgroup of the adult Swedish volunteers who had received ETVAX alone or together with dmLT and an additional group of 22 age-matched naive Swedish adults received a single dose of ETVAX 1–2 years after the primary vaccinations [9]. The vaccine was administered in bicarbonate buffer in a volume of 150 mL; placebo recipients were given the same volume of buffer alone.

#### Children

ETVAX ± dmLT adjuvant or placebo was administered in 2 doses to 3 groups of Bangladeshi children 24–59 months (n = 125; B cohorts), 12–23 months (n = 97; C cohorts), and 6–11 months of age (n = 158; D cohorts), given fractionated (one eighth, one fourth, or one half) amounts of a full vaccine dose [7]. The vaccine was given alone or together with 2.5, 5, or 10 µg of dmLT adjuvant suspended in bicarbonate buffer in volumes of 10–30 mL depending on age. Placebo recipients received the buffer adjusted to the same volumes as vaccine recipients for each age group. Vaccine and placebo were given in a double-blind manner 2 weeks apart, and immune responses were recorded before (day 0) and then 5 days (day 19) and 28 days (fecal responses only) after the second dose.

### Immune Responses

Mucosal immune responses were assessed as antibody-secreting cell (ASC) responses, determined as antibody in lymphocyte secretions (ALS) in cultures of peripheral blood mononuclear cells (PBMCs) and secretory IgA (SIgA) responses in fecal extracts against CFA/I, CS3, CS5, CS6, heat-labile toxin B-subunit (LTB), and O78 lipopolysaccharide (LPS) [6, 7, 8] (Svennerholm A-M, Qadri F, Lundgren A, et al., 2021, submitted for publication). Studies were also undertaken to evaluate whether ETVAX could induce mucosal immune responses against cross-reactive CF antigens, ie, the CFA/I-related CS1, CS14, and CS17 and the CS5-related CS7 [10, 11].

The capacity of ETVAX to induce immunological memory was studied in adult Swedes given a single booster dose of ETVAX 1–2 years after priming vaccinations and for comparison a group of naive Swedes given a single dose of ETVAX [9]. Immune responses were assessed as ALS responses in PBMCs isolated before (day 0) and 4 or 5 and 7 days after the single vaccine dose.

The ALS specimens from the Swedish subjects were analyzed by enzyme-linked immunosorbent assay (ELISA), whereas ALS responses in the Bangladeshi subjects were evaluated by an electrochemiluminescence assay using the Mesoscale Discovery Platform (MSD) [8]. The SIgA immune responses in fecal extracts were determined as the specific SIgA titer against each antigen divided by the total SIgA concentration of the sample by ELISA [6, 7].

## RESULTS

### Safety

All studies of ETVAX in adults have shown that the vaccine is safe and well tolerated with no significant differences in adverse events between vaccinees and placebo recipients [6, 8, 9]. In children, adverse events were few and mild, the most common being vomiting, which was related to dose and age [7]. No serious adverse events related to the vaccine were observed in any of the volunteers.

### Mucosal Immune Responses

**Figure 1.**
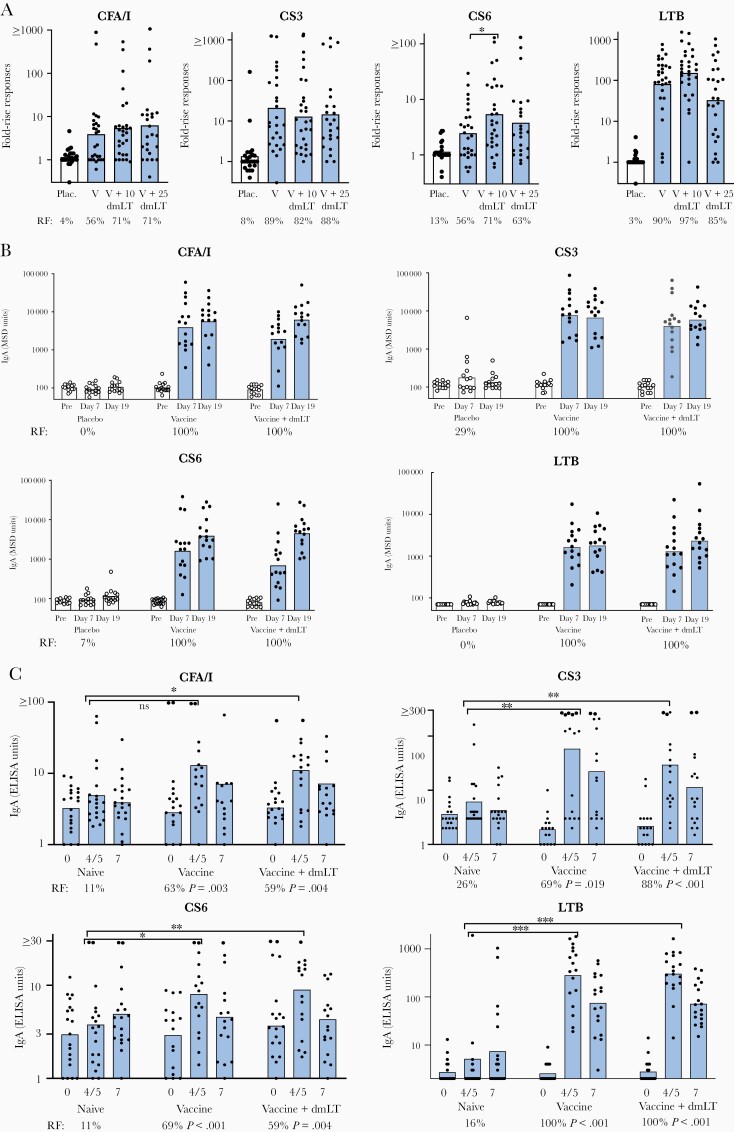
Antibody in lymphocyte secretion (ALS) IgA immune responses against ETVAX colonization factors (CFs) and LTB in (A) Swedish adults given 2 biweekly doses of ETVAX alone or ETVAX + 10 or 25 µg of double-mutant heat-labile toxin (dmLT) or placebo; (B) Bangladeshi adults given 2 biweekly doses of ETVAX alone or ETVAX + 10 µg of dmLT or placebo; and (C) Swedish adults given a single dose of ETVAX, including subjects who 1–2 years earlier had been given 2 doses of ETVAX alone or ETVAX + 10 µg of dmLT, and naive age-matched Swedish adults who previously had not been immunized with ETVAX. (A–C) Immune responses were determined as ≥2-fold rises in ALS antibody levels between pre- and postimmunization specimens collected on day 7 after the first day and 5–7 days (day 19–21) after the second dose or on days 4 or 5 and 7 after the single booster dose. Fold-rise responses are shown as geometric mean (bars) responses against the respective vaccine antigen for each study group; dots indicate individual responses; fold-rises in Figure 1A were determined as the maximal response after the first or second dose. Responder frequencies (RF) in vaccine and placebo recipients are indicated below the bars. Based on results from [6, 8, 9].

In the initial studies in Swedish adults, 2 oral immunizations with ETVAX ± dmLT were shown to induce significant ALS immune responses against all the CFs and LTB in a majority of the volunteers (56%–90% against vaccine alone and 58%–97% against vaccine + dmLT) (Figure 1A) (results against CS5, data not shown). Administration of ETVAX + 10 µg of dmLT adjuvant resulted in significantly higher immune responses than vaccine alone against the antigen present in lowest amount in the vaccine, CS6. The same vaccination schedule in adult Bangladeshis induced significant ALS responses against all the vaccine antigens in 100% of the vaccinees, but no enhancement by the adjuvant was recorded (Figure 1B).

The study in Swedish adults to evaluate the capacity of ETVAX to induce immunological memory revealed significantly stronger and earlier appearing ALS responses against all of the 5 primary vaccine antigens in volunteers previously vaccinated with ETVAX + dmLT compared with naive individuals given a single dose of vaccine (Figure 1C). Most of the subjects previously vaccinated with ETVAX alone also responded stronger than the naive subjects to the single vaccine dose.

**Figure 2.**
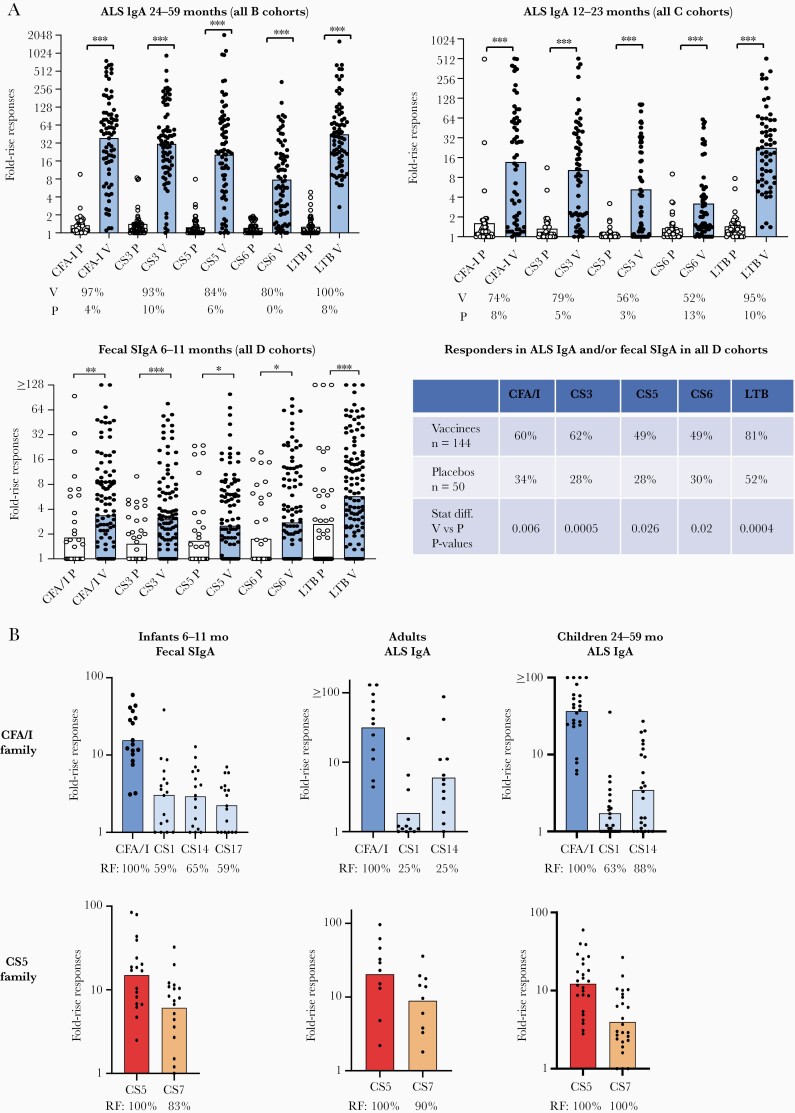
(A) Mucosal immune responses in Bangladeshi children and infants given 2 biweekly doses of ETVAX ± double-mutant heat-labile toxin (dmLT) against colonization factors (CFs) and LTB: (B cohorts) antibody in lymphocyte secretion (ALS) IgA response in children 24–59 months and (C cohorts) 12–23 months; (D cohorts) fecal secretory IgA (SIgA)/total SIgA response in infants 6–11 months and (table) responder frequencies (RFs) in ALS IgA and/or fecal SIgA/total SIgA in the infants. Immune responses were determined as ≥2-fold rises in ALS titers or SIgA/total SIgA levels between pre- and postimmunization specimens collected on day 7 after the first day and 5 days (day 19) after the second dose. Fold-rise responses, determined as the maximal response after the first or second dose, are shown as geometric mean responses (bars) against the respective vaccine antigen for each study group; dots indicate individual responses. The RFs in vaccine (V) and placebo recipients (P) are indicated below the bars and in the table. Based on results from [7]. (B) Cross-reactive IgA immune response against CFA/I and CS5-related CFs in ALS specimens collected from Bangladeshi adults and children 24–59 months and SIgA/total SIgA responses in fecal specimens from 6- to 11-month-old infants. Paired pre- and postimmunization specimens from subjects previously shown to have responded to CFA/I [7, 8] were tested for immune responses against corresponding concentrations of CS1, CS14, and for infants (fecal specimens) also against CS17 and specimens from subjects who had previously responded to CS5 [7, 8] were similarly tested for responses against CS7. Fold-rise responses are shown as geometric means (bars). The RFs are indicated below the bars.

The studies of mucosal immune responses in Bangladeshi children showed highly significant ALS responses to all the CFs and LTB in 80%–100% of the 24- to 59-month-old toddlers and in 52%–95% of the 12- to 23-month-old children given either one-fourth or one-half dose of ETVAX ± dmLT (Figure 2A). Only 15%–40% of the 6- to 11-month-old infants given one-eighth or one-fourth dose ± 2.5 µg of dmLT or one-half dose of ETVAX alone mounted ALS responses to the CFs; ALS responses against CS5 and CS6 were not significantly higher than in the placebos (data not shown). However, high and significant SIgA immune responses were observed against all of the CFs and LTB in fecal extracts from the infants (Figure 2A). Infants receiving one-fourth dose of ETVAX + 2.5 µg of dmLT responded already to the first vaccine dose in feces and significantly better than to a single one-fourth dose of ETVAX alone [7].

Because 3 of the ETVAX strains were based on O78 *E coli* bacteria, mucosal immune responses against O78 LPS were evaluated in the Bangladeshi children (Svennerholm A-M, Qadri F, Lundgren A, et al., 2021, submitted for publication). These analyses showed that the children 12–59 months of age also mounted highly significant ALS responses against O78 LPS (data not shown). As observed for immune responses against the CFs, ALS responses to the LPS antigen in infants were infrequent (less than 10% had increased ALS antibody levels after vaccination). However, highly significant SIgA responses against O78 LPS were recorded in fecal extracts, and those responses were significantly enhanced, both with regard to magnitudes and frequencies, by addition of dmLT adjuvant (data not shown).

### Immune Responses Against Cross-Reactive Antigens

The ETEC CFs are heterogenous, but there are certain CFs that are immunologically related, eg, CFs belonging to the CFA/I and CS5 families [10]. We have previously shown that ETVAX could induce cross-reactive mucosal immune responses against CFs in adult Swedes [11]. Hence, we analyzed ALS as well as fecal specimens from the Bangladeshi adults and children who had responded to CFA/I and CS5, respectively, for cross-reactive immune response. A majority of the adults and children who had developed ALS responses against CFA/I had also responded to other members of the CFA/I family, in particular CS14 and CS1, and infants had developed cross-reactive immune responses against CS1, CS14, and CS17 in feces. Cross-reactive immune responses were even more prevalent and higher against CS7 in those vaccinees who had responded to CS5 (Figure 1C) (Leach S, unpublished observations, 2020).

## Discussion

We developed an oral ETEC vaccine consisting of inactivated ETEC bacteria expressing prevalent CFs and recombinantly produced CTB in the 1990s [3, 12]. This vaccine was found to be safe and immunogenic in adults in different nonendemic countries, as well as in children and adults in Bangladesh [3]. The vaccine also conferred protection against moderate/severe diarrhea in American travelers to Mexico-Guatemala [12]. However, the protective efficacy of the vaccine was not significant in young Egyptian children and a full vaccine dose induced vomiting in many of the children [3]. Therefore, we developed a second-generation ETEC vaccine consisting of recombinant *E coli* strains expressing increased amounts of the most prevalent CFs and given together with an alternative toxoid, LCTB*A*, which induced stronger immune responses against LT than CTB [4]. This modified multivalent ETEC vaccine, ETVAX, has been extensively tested for safety and immunogenicity, initially in different Phase 1 trials in adult Swedish volunteers and subsequently in Phase 1/2 studies in adults and descending age groups of children in Bangladesh (Table 1). These studies have shown that the vaccine is safe and immunogenic in a full dose in adults and in fractionated doses in children and infants [6, 7–9]. Based on these results, the vaccine was recently evaluated for safety, immunogenicity, and protective efficacy in Finnish travelers to Benin in West Africa [13] and for safety and immunogenicity in adults and children 6–23 months of age in Zambia [14].

**Table 1.** Clinical Trials of Second-Generation Oral-Inactivated ETEC Vaccines^a^

Trial	Vaccine	Volunteers	Publication
OEV-120, Phase 1, Sweden	Prototype vaccine (recombinant CFA/I strain + LCTB*A*) compared with first-generation ETEC vaccine (inactivated ETEC bacteria + CTB)	59 Swedish adults: 2 doses	Ref. [18]
OEV-121, Phase 1, Sweden	4 recombinant strains expressing CFA/I, CS3, CS5, CS6 (multivalent vaccine: 10^11^ bacteria + 1 mg LCTB*A*) ±10 or 25 µg dmLT	129 Swedish adults: 2 doses	Ref. [6]
OEV-121A, Phase 1, Sweden	Booster with multivalent ETEC vaccine	60 Swedish naive and previously immunized adults: 1 dose	Ref. [9]
OEV-122, Phase I/II, Bangladesh	Adults: ETVAX (multivalent ETEC vaccine ±10 µg dmLT) Children: fractionated doses of ETVAX ±dmLT	45 Bangladeshi adults: 2 doses 450 Bangladeshi children, 6–59 months: 2 doses	Ref. [8] Ref. [7]
OEV-123, Phase 2b, Benin	ETVAX (multivalent vaccine including 10 µg of dmLT)	729 Finnish adult travelers: 2 doses	Kantele et al [13]
OEV-124, Phase 1, Zambia	Adults: ETVAX (multivalent vaccine including 10 µg of dmLT), full dose Children: ETVAX, 1/4 or 1/8 dose	40 Zambian adults: 1 dose 229 children, 6–23 months: 2 initial doses + a booster	Sukwa et al [14]

Abbreviations: CTB, cholera toxin B subunit; dmLT, double-mutant heat-labile toxin; ETEC, enterotoxigenic *Escherichia coli*.

^a^The different studies were conducted during the period 2010–2021.

The vaccine trials reviewed here (Table 1) have aimed to evaluate the capacity of ETVAX to induce mucosal immune responses against the main putative protective antigens of ETVAX, the CFs, LTB, and O78 LPS in different populations, ie, adults living in highly ETEC-endemic as well as a nonendemic countries and, more importantly, in young children and infants living in ETEC-endemic areas who are the main target groups for an ETEC vaccine [1, 7]. The aim was also to evaluate whether the mucosal adjuvant dmLT [5] is capable of enhancing immune responses against ETVAX antigens, as well as serve as a model for the capacity of the adjuvant to enhance immune responses also against antigens in other oral enteric vaccines, eg, *Shigellae*, *Salmonellae*, and cholera vaccines.

Our studies have shown that ETVAX induced significant mucosal, ALS, or fecal, immune responses against all of the different vaccine antigens in all populations studied. Immune responses were clearly age-dependent, as demonstrated in the Bangladeshi trial with the highest and most prevalent immune responses in adults and older children and decreasing magnitudes and frequencies of responses in younger children and infants [7, 8]. It is interesting to note that, whereas ALS responses against both the CFs and O78 LPS were low and infrequent in infants, fecal immune responses in this age group were comparable to the ALS responses in older children. This may suggest that determination of ASC or ALS responses is not suitable for studies of mucosal immune responses in infants, as has also been shown for an oral rotavirus vaccine [15].

Our studies also showed that dmLT adjuvant could enhance mucosal immune responses against ETVAX, in particular against weakly immunogenic antigens, eg, CS6 in ALS specimens from Swedish vaccinees and CFs and O78 LPS in feces from infants [6, 7]. These findings may support usage of the adjuvant to enhance the mucosal immunogenicity of other oral vaccines, in particular in nonprimed populations and infants.

ETVAX was also capable of inducing significant immunological memory against both the CFs and LTB that persisted for at least 1–2 years. We have previously shown that CTB in an oral cholera vaccine could induce a mucosal immunological memory for at least 10 years [16] and hence the memory induced by ETVAX may remain for a considerably longer period than 2 years. A longer duration of the memory induced by ETEC antigens is supported by the comparatively low incidence of ETEC disease in endemic areas in children above 5 years of age who have been naturally primed by repeated ETEC infections during early childhood [1, 17].

Due to the large heterogeneity of ETEC CFs, it has been important to evaluate whether ETVAX, which only contains the 4 most prevalent CFs, could also induce immune responses against related CFs. We have previously shown that ETVAX induced both ALS and fecal immune responses against CFA/I-related and CS5-related CFs in Swedish adults [11]. We have now confirmed that ETVAX may also induce such cross-reactive immune responses in Bangladeshi children and adults, suggesting increased protective coverage of the vaccine.

Based on the promising findings described above, a Phase 2b study has been initiated in infants and young children in the Gambia, to evaluate the capacity of 2 initial doses of ETVAX followed by a booster dose 3 months later to induce protection against ETEC diarrhea. The selection of vaccine dose for this trial, ie, one-fourth dose of ETVAX + 2.5 µg of dmLT, has been based on the results from the previous studies described.

Limitations of these studies include difficulties in evaluating mucosal immune responses against ETVAX in infants by the most commonly used approaches, ie, determination of ASCs in the circulation by enzyme-linked immunospot (ELISPOT) or ALS methods. Instead, we determined SIgA responses in fecal extracts in this age group by rather complex and time-consuming procedures. It is unfortunate that determination of fecal antibody responses in older children and adults in the ETEC-endemic countries chosen for these studies was not possible, due to difficulties in retrieving fecal extracts with sufficient levels of SIgA from older subjects. The study groups receiving each vaccine and adjuvant dose combination were relatively small, limiting the possibilities to detect small differences in immunogenicity between groups, particularly in younger age groups with more frequent natural ETEC infections.

A current obstacle for initiating Phase 3 studies of the protective efficacy of ETVAX both in travelers and children in endemic areas is the ongoing coronavirus disease 2019 (COVID-19) pandemic, which has led to practical challenges associated with research and which promotes production and testing of COVID-19 vaccines, resulting in decreased engagement by vaccine testing units and regulatory authorities to engage in trials testing diarrheal vaccine candidates.

## Conclusions

We conclude that the good safety profile and significant mucosal immune responses induced by ETVAX against all key protective antigens in all age groups strongly support further testing of the vaccine in Phase 3 trials in travelers as well as in young children in ETEC-endemic areas. Phase 2b studies in children are currently ongoing in the Gambia until 2023, and they are in late stages of preparation in Zambia. It is our hope that ETVAX may an important tool to control one of the most common causes of diarrhea worldwide in the future.

## Notes

***Acknowledgments.*** We gratefully acknowledge the support of the study participants and their parents and the dedicated field workers and personnel at International Centre for Diarrhoeal Disease Research, Bangladesh (icddr,b) as well as the laboratory personnel at University of Gothenburg.

***Author contributions.*** A.-M. S., A. L., S. L., and F. Q. designed the studies. A.-M. S. and A. L. wrote the manuscript. All authors contributed to the interpretation of results and critical review and revision of the manuscript and have approved the final version.

***Financial support.*** This work was funded by PATH (Grant Number OPP1112376); the United Kingdom (UK)’s Foreign and Commonwealth Development Office (Grant Number 204139–101); The Swedish Research Council and the Swedish Foundation for Strategic Research (Grant Number SB12-0072). The icddr,b gratefully acknowledges the following donors who provided unrestricted support: Governments of Bangladesh, Canada, Sweden, and the UK.

***Potential conflicts of interest.*** A.-M. S. is a shareholder of the biotech company Gotovax AB that may receive a small royalty on sales of ETVAX if it becomes a commercial product. S. L. has received consultancy fees from ETVAX AB, the developer of ETVAX. All authors have submitted the ICMJE Form for Disclosure of Potential Conflicts of Interest. Conflicts that the editors consider relevant to the content of the manuscript have been disclosed.
